# Development and validation of muscle strength improvement in 24 hours to predict ischemic stroke patients’ outcome after endovascular therapy: A retrospective cohort study

**DOI:** 10.1097/MD.0000000000042055

**Published:** 2025-06-06

**Authors:** Zile Yan, Mohammad Mofatteh, Thanh N. Nguyen, Robert W. Regenhardt, Xuehua Zeng, Suzuki Dharmarasu, Jiale Wu, Baoxin Chen, Zhiyi Zeng, Jianfang Gao, Rongshen Yang, Shuiquan Yang, Xuxing Liao

**Affiliations:** a Department of Neurology and Advanced National Stroke Center, Foshan Sanshui District People’s Hospital, Foshan, Guangdong Province, China; b School of Medicine, Dentistry and Biomedical Sciences, Queen’s University Belfast, Belfast, United Kingdom; c Department of Neurology, Radiology, Boston University Chobanian & Avedisian School of Medicine, Boston, MA, USA; d Department of Neurology, Neurosurgery, Massachusetts General Hospital, Harvard Medical School, Boston, MA, USA; e Department of Research and Education, Foshan Sanshui District People’s Hospital, Foshan, Guangdong Province, China; f Department of Medicine, Yong Loo Lin School of Medicine, National University of Singapore, Singapore, Singapore; g School of Medicine, Shaoguan University, Shaoguan, Guangdong Province, China; h Department of Neurology and Advanced National Stroke Center, Foshan Sanshui District People’s Hospital, Foshan, Guangdong Province, China; i School of Humanities and Social Sciences, Macao Polytechnic Institute, Macao, China; j Department of Neurosurgery and Advanced National Stroke Center, Foshan Sanshui District People’s Hospital, Foshan, Guangdong Province, China.

**Keywords:** endovascular therapy, ischemic stroke, modified Rankin scale, MRC scale, muscle strength, patient outcome

## Abstract

The relationship between muscle strength improvement and ischemic stroke outcome following endovascular therapy (EVT) for emergency treatment of patients has not been well studied. We developed the prognostic value of the change in muscle strength scores on the Medical Research Council (MRC) scale within 24 hours of EVT as a predictor of EVT outcomes. We retrospectively analyzed prospectively collected data from 142 consecutive patients with ischemic stroke who underwent EVT between August 2018 and February 2022 at a comprehensive stroke center. Early muscle strength improvement (EMSI) was defined as 3 levels of muscle strength improvement on the MRC scale or improvement to grade 4 or 5 of lower-limb muscle strength (LLMS) 24 ± 8 h after EVT. A favorable outcome was defined as a 3-months outcome of the modified Rankin scale (mRS) 0% to 2. 84.2% of EMSI patients had a favorable outcome, in comparison to only 21.2% of non-EMSI patients (*P* < .001). Similarly, 47.1% of patients died at 3-months in the non-EMSI group, while a significantly lower mortality (8.8%) was observed in the EMSI group (*P* < .001). LLMS 24 hours post-EVT was significantly associated with 90-day outcomes (*P* < .001). Early muscle strength improvement in the emergency setting may be a useful predictor of a desired functional outcome in patients with large-vessel occlusion stroke following EVT.

## 1. Introduction

Mechanical thrombectomy has been shown to improve outcomes in patients with acute ischemic stroke with large-vessel occlusion (LVO) up to 24 hours after stroke onset.^[1,2]^ Peripheral muscle strength may be an important predictor of patient prognoses.^[3]^ In critically ill patients, poor muscle strength is indicative of a higher risk of reintubation, in-hospital mortality, and poor functional outcomes.^[3]^ Muscle strength has been used to predict loss of functional status in hospitalized patients, and lower muscle strength has been associated with postoperative morbidity.^[4]^ Impaired leg muscle strength has also been used as a marker of frailty and to independently predict clinical outcomes in patients with acute heart failure.^[5]^ Weakness or loss of lower-limb muscle strength (LLMS) as a result of stroke has a poor influence on dynamic balance and is associated with patient outcomes.^[6]^

Previous LVO studies demonstrated that early neurological improvement could predict outcomes after endovascular therapy (EVT).^[7,8]^ Clinical assessment of motor impairment within a few days of stroke can help predict subsequent recovery.^[9]^ Indeed, recovery of motor function after ischemic stroke is important for regaining independence from ambulation and activities of daily living. In this study, we aimed to develop the prognostic value of change in muscle strength scores on the Medical Research Council (MRC) Scale within 24 ± 8 hours of EVT as a potential predictor of EVT outcomes. To our knowledge, this is the first study to evaluate early muscle strength improvement (EMSI) as a predictor of long-term outcomes after EVT.

## 2. Materials and methods

### 2.1. Study population and inclusion criteria

This retrospective study analyzed prospectively collected data of 142 consecutive stroke patients with LVO who underwent EVT from the inception of EVT services at our center from August 2018 to February 2022 at Foshan Sanshui District People’s Hospital in China. The Foshan Sanshui District People’s Hospital is the only comprehensive stroke center to perform EVT in the Foshan Sanshui District, where there are more than 800,000 inhabitants. The data were derived from the Big Data Observatory Platform for Stroke in China and the hospital data platform. The inclusion criteria for this study were as follows: EVT for patients with LVO treated within 24 hours of symptom onset; age ≥ 18 years; and acute occlusion of the basilar artery, internal carotid artery, or M1 to M2 segments of the middle cerebral artery or other large cerebral vessels. Patients with missing follow-up data were excluded from this study.

### 2.2. Outcome variables

The following data were collected: age, sex, vascular risk factors (such as hypertension, diabetes, and atrial fibrillation), initial premorbid modified Rankin scale (mRS), National Institute of Health Stroke Scale Score (NIHSS), muscle strength scale of the upper and lower limbs before and after EVT, door-to-needle time (DNT), onset-to-needle time (ONT), door-to-puncture-time (DPT), last-known normal-to-puncture-time (LKNPT), door-to-recanalization time (DRT), and modified thrombolysis in cerebral infarction (mTICI) after thrombectomy. Successful reperfusion was defined as an mTICI ≥ 2b.

### 2.3. Muscle strength measures

The (standard) MRC scale for muscle strength/weakness was used to measure muscle strength.^[10]^ A stroke nurse or neurologist measured and recorded bilateral limb muscle strength every 4 hours for at least 24 hours after EVT, as required by Advanced National Stroke Centers and first-degree nursing. Hemiplegia muscle strength was recorded in this study. If the patient had bilateral acute weakness, the weaker limb was used for the analyses. For patients discharged to the hospice within 24 hours, muscle strength at discharge was recorded.

### 2.4. Early muscle strength improvement definition

In this study, we defined EMSI as 3 levels of muscle strength improvement on the MRC scale compared to the level at presentation before EVT or improvement to grade 4 or 5 of LLMS at 24 ± 8 h after EVT.

### 2.5. Outcome measures

Patient outcomes were evaluated using the mRS at 3-months after EVT. The mRS was followed up routinely by stroke nurses and neurologists via telephone or in-person consultations during the 3-month outpatient follow-up. A favorable outcome was defined as an mRS of 0 to 2, whereas an unfavorable outcome was defined as an mRS of 3 to 6.

### 2.6. Ethics

This study was approved by the Foshan Sanshui District People’s Hospital Review Board. All methods were performed in accordance with the relevant guidelines and regulations of Foshan Sanshui District People’s Hospital. Written informed consent was obtained from the participant’s legal guardian/next of kin to perform the EVT.

### 2.7. Statistical analysis

Non-normally distributed continuous data were reported as medians with an inter-quartile range. The nonparametric Mann–Whitney *U* test was used, as appropriate. Normally distributed data were reported as mean ± standard deviation. Student *t* test was used for comparisons as appropriate. Results were considered statistically significant if the *P*-value was <.05. Analyses of the area under the receiver operating characteristic curve (AUC) were performed to estimate the ability of the lower and upper limb muscle strength 24 hours after EVT to predict long-term outcomes. IBM SPSS version 26 (IBM Corp., Armonk) was used for the statistical analyses.

## 3. Results

The baseline patient characteristics are presented in Table 1. Our study included 146 patients, of whom 4 were excluded because they were lost to follow-up and were not included in our analyses. In total, 142 patients underwent EVT and were divided into 2 groups: 57 and 85 patients in the EMSI and non-EMSI groups, respectively. The pre-EVT median NIHSS was 13.00 (IQR 10.00, 17.00) in the EMSI group, and 19.00 (15.00, 22.00) in the non-EMSI group (*P* < .001). Similarly, the EMSI group had a shorter DNT time of 127.00 (104.00, 186.50) minutes compared to 221.00 (158.50, 299.50) minutes in the non-EMSI group (*P* < .001). More successful recanalization (mTICI ≥ 2b) was achieved in the EMSI group (98.24%) than in the non-EMSI group (77.64%) (*P* < .001). There were no significant differences in age, sex, hypertension, diabetes, chronic kidney disease, smoking status, pre-EVT Alberta Stroke Program Early CT Score (ASPECTS), pre-EVT mRS, intravenous (IV) thrombolysis rate, occlusion sites, ONT, DPT, DRT, and LKNPT between the 2 groups.

**Table 1:** Comparison of baseline characteristics of EMSI and non-EMSI at 24 hours after EVT.

Characteristic	EMSI (N = 57)	Non-EMSI (N = 85)	χ^2^/*t*/*z*	*P*
Age mean ± SD	63.87 ± 12.31	66.00 ± 13.73	0.940	.349
Male, n, %	41 (71.93)	59 (69.41)	0.104	.747
Hypertension, n, %	31 (54.38)	51 (60.00)	0.441	.507
Diabetes mellitus, n, %	12 (21.05)	20 (23.52)	0.120	.729
Chronic artery disease, n, %	7 (12.28)	22 (25.88)	3.884	.049
Prior stroke, n, %	12 (21.05)	19 (22.35)	0.034	.854
Chronic kidney disease, n, %	7 (12.28)	10 (11.76)	0.009	.926
Atrial fibrillation, n, %	20 (35.08)	36 (42.35)	0.754	.385
Smoker, n, %	20 (35.08)	18 (21.17)	3.369	.066
Median NIHSS pre-EVT (IQR)	13.00 (10.00, 17.00)	19.00 (15.00–22.00)	4.964	<.001
ASPECTS pre-EVT (IQR)	9.00 (8.00, 9.00)	8.00 (8.00–9.00)	1.488	.137
IV thrombolysis, n, %	24 (42.10)	44 (51.76)	1.276	.259
Admission upper muscle strength	1.00 (0.00,3.00)	1.00 (0.00–1.00)	−3.028	.002
Admission lower muscle strength	2.00 (1.00, 3.00)	1.00 (1.00–2.00)	−4.108	<.001
DNT (IQR)	34.00 (28.00, 42.25)	50.50 (34.75–63.00)	3.310	.001
ONT (IQR)	121.00 (108.50, 213.00)	140.00 (110.00–182.00)	0.222	.825
Occlusion sites				
Distal/terminal ICA n, %	6 (10.52)	15 (17.64)	9.753	.083
MCA-M1 n, %	33 (57.89)	29 (34.11)		
MCA-M2 n, %	0 (0.00)	2 (2.35)		
Tandem n, %	11 (19.29)	20 (23.52)		
Basilar n, %	7 (12.28)	17 (20.00)		
Others n, %	0 (0.00)	2 (2.35)		
DPT (IQR), min	127.00 (104.00–186.50)	133.00 (104.50–199.50)	0.327	.744
DRT (IQR), min	199.00 (156.00–256.00)	221.00 (158.50–299.50)	1.301	.193
LKNPT (IQR), min	299.00 (210.00–505.50)	290.00 (197.50–470.00)	0.233	.816
mTICI post ≥ 2b, n, %	56 (98.24)	66 (77.64)	11.964	.001

DPT = door-to-puncture-time, DRT = door-to-recanalization time, EMSI = early muscle strength improvement, EVT = endovascular therapy, IQR = inter-quartile range, LKNPT = last-known normal-to-puncture-time, mTICI = modified thrombolysis in cerebral infarction, NIHSS = National Institute of Health stroke scale/score, ONT = onset-to-needle time.

The outcomes of the EMSI and non-EMSI groups at 24 hours after EVT are shown in Table 2. Fewer lung infections (*P* < .001) and sICH (*P* = .002) were observed in the EMSI group. The in-hospital mortality/hospice discharge rates were 1.75% in the EMSI group and 34.11% in the non-EMSI group (*P* < .001). 84.21 Of the patients in the EMSI group, 84.21% had favorable outcomes compared with 21.17% of patients in the non-EMSI group (*P* < .001). Furthermore, 47.05% of the patients in the non-EMSI group died at 3 months, while only 8.77% died in the EMSI group (*P* < .001).

**Table 2:** Comparison of outcome of EMSI and non-EMSI in 24 hours after EVT.

Outcomes	EMSI (N = 57)	Non-EMSI (N = 85)	X^2^/t/z	*P*
Lung infection, n, %	5 (8.77)	43 (50.58)	26.663	<.001
Urinary infection, n, %	5 (8.77)	5 (5.88)	0.106	.745
sICH, n, %	1 (1.75)	16 (18.82)	9.433	.002
mRS discharge (IQR)	2.00 (2.00)	5.00 (1.00)	8.225	<.001
In-hospital Mortality/hospice discharge home, n, %	1 (1.75)	29 (34.11)	21.446	<.001
3-mo favorable outcome, n, %	48 (84.21)	18 (21.17)	54.497	<.001
Mortality at 3 mo, n, %，	5 (8.77)	40 (47.05)	23.105	<.001
Poor outcome, n, %	9 (15.78)	67 (78.82)	54.497	<.001

Favorable outcome: mRS (0–2) at 3 mo. Poor outcome: mRS ≥ 3.

EMSI = early muscle strength improvement, EVT = endovascular therapy, IQR = inter-quartile range; sICH = symptomatic intracranial hemorrhage.

**Figure 1.**
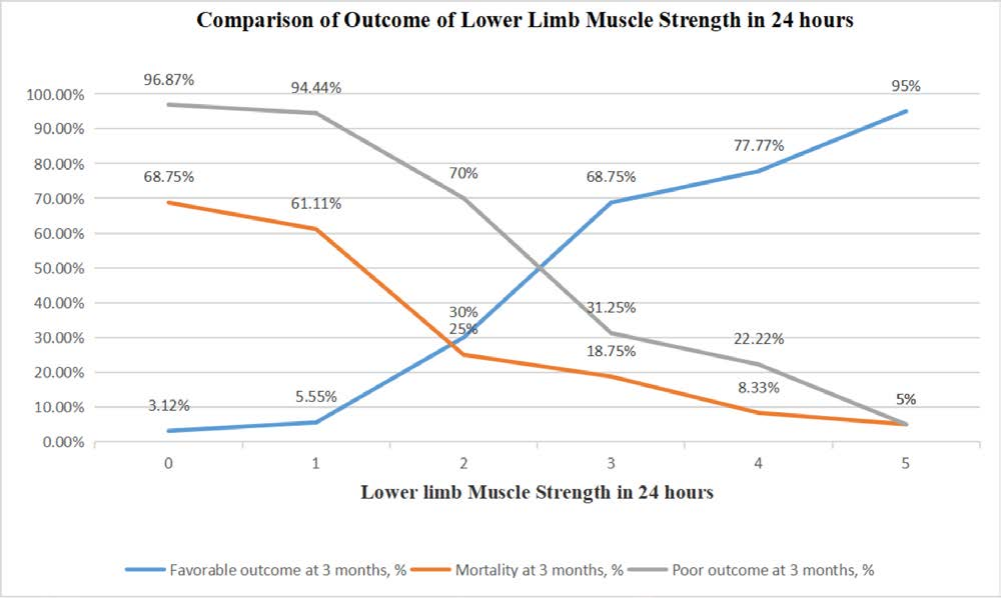
Comparison of outcomes for patients with different LLMS scores 24 hours after EVT. EVT = endovascular therapy, LLMS = lower-limb muscle strength.

The outcomes of LLMS 24 hours after EVT are shown in Table 3 and Figure 1. LLMS 24 hours after EVT was associated with long-term clinical outcomes; more patients achieved a favorable 90-day outcome, and mortality decreased as 24-hour muscle strength increased. Patients with values corresponding to LLMS (0, 1, 2, 3, 4, 5) 24 hours after EVT achieved a 90-day favorable outcome in 3.12%, 5.55%, 30.00%, 68.75%, 77.77%, and 95.00% of cases, respectively.

**Table 3:** Comparison of outcomes for patients with different LLMS scores 24 hours after EVT.

	LLMS = 0	LLMS = 1	LLMS = 2	LLMS = 3	LLMS = 4	LLMS = 5	*P*
In-hospital mortality/hospice discharge home, n, %	20 (62.50)	5 (27.77)	2 (10.00)	2 (12.50)	1 (2.77)	0 (0.00)	<.001
3-mo favorable outcome, n, %	1 (3.12)	1 (5.55)	6 (30.00)	11 (68.75)	28 (77.77)	19 (95.00)	<.001
Mortality at 3 mo, n, %	22 (68.75)	11 (61.11)	5 (25.00)	3 (18.75)	3 (8.33)	1 (5.00)	<.001
The poor outcome, n, %	31 (96.87)	17 (94.44)	14 (70.00)	5 (31.25)	8 (22.22)	1 (5.00)	<.001

EVT = endovascular therapy, LLMS = lower-limb muscle strength.

**Figure 2.**
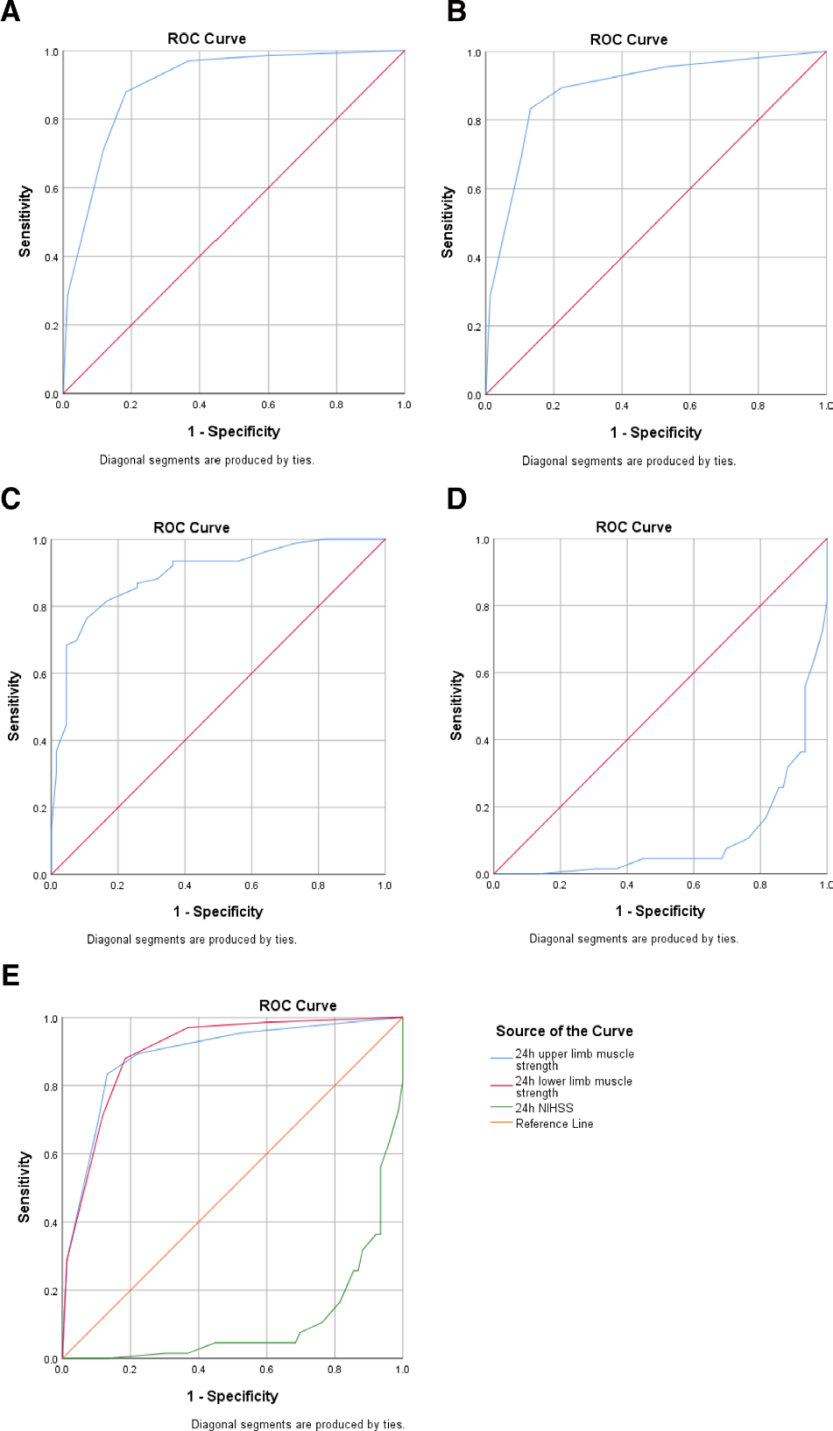
Receiver operating characteristic curve of muscle strength in 24 h predicting patient prognosis.

The receiver operating characteristic curve of upper and LLMS 24 hours after EVT for prediction of a 3-month favorable outcome (mRS of 0–2) is shown in Figure 2. The area under the curve of LLMS 24 hours after EVT for predicting 3-month favorable outcome was 0.901 (95% CI = 0.849–0.952; *P* < .001). The area under the curve of upper limb muscle strength 24 hours after EVT for predicting 3-month favorable outcome was 0.890 (95% CI = 0.833–0.946; *P* < .001). The area under the curve of NIHSS 24 hours after EVT for predicting a 3-month unfavorable outcome was 0.893 (95% CI = 0.849–0.946; *P* < .001) and a favorable outcome was 0.107 (95% CI = 0.054–0.160; *P* < .001).

## 4. Discussion

Although EVT improves patient outcomes, the significance of muscle strength after treatment in determining long-term outcomes has not been well studied. The results of our study suggest that EMSI can predict the outcome of patients with LVO stroke following EVT. Both upper and LLMS had similar predictive capabilities in our receiver operating characteristic analyses. More patients achieved favorable outcomes and suffered less mortality as muscle strength increased 24 hours after EVT.

The most important predictive factors for limb recovery following stroke may be the initial severity of motor impairment and initial level of disability.^[11]^ A previous study demonstrated that early functional recovery of muscle strength 72 hours after stroke onset could predict long-term outcome.^[12]^ Another study demonstrated that admission MRC motor strength grade could be a robust predictor of mortality in patients with acute ischemic stroke undergoing EVT.^[13]^ Our study expands upon these results, supporting the idea that EMSI is an important determinant of 90-day outcomes.^[14,15]^

Successful and early mechanical thrombectomy in ischemic stroke patients is associated with rapid alleviation or reversal of neurological deficits, resulting in better patient outcomes.^[16–18]^ However, despite treatment, patients can suffer hemorrhagic transformation, and EVT is sometimes unsuccessful in recanalizing the occlusion. Indeed, both have been associated with neurological worsening.^[8]^ Our study showed that, compared to the EMSI group, the non-EMSI group had more sICH and a less successful recanalization rate.

Acute ischemic stroke is a dynamic disease; patients often experience fluctuations in symptoms or deficits based on the degree of viable penumbra, collateral patterns, or extent of perfusion.^[19]^ Indeed, there is a higher likelihood of early changes in the disease course.^[20]^ Early neurological improvements have previously been studied and defined by the absolute or relative change in the NIHSS score after mechanical thrombectomy compared to the baseline NIHSS.^[7,8,16]^ Muscle strength is an important component of the National Institutes of NIHSS used to assess neurological improvement. A previous study suggested that NIHSS 24 hours after mechanical thrombectomy may be predictive of 90-day mRS 0 to 2, with an area under the curve of 0.885,^[16]^ and similarly, our study showed that NIHSS at 24 hours predicts 90-day outcome, with an area under the curve of 0.893. The area under the curve of our study assessing the predictive ability of LLMS was comparable at 0.901, suggesting that a muscle strength recovery prediction model may also predict EVT outcomes similar to the 24-hour NIHSS early neurological improvement endpoint.

Upper and LLMS assessments have other advantages over NIHSS. The NIHSS is a 15-item examination tool that can take nearly 8 minutes to administer by trained personnel. Furthermore, assessors are required to undergo NIHSS training to accurately score subtle variations in clinical presentation.^[21]^ It also has several intricate features and requires understanding of aphasia, neglect, and other aspects of neurological examination. The advantage of muscle strength assessment is that it is convenient, simple, and routinely performed by healthcare providers in clinical practice. Early muscle strength assessment can be a practical quantitative surrogate for predicting ischemic patient outcomes after EVT.

EMSI has the potential to be integrated into stroke care pathways as a rapid, bedside assessment tool for prognostication. The simplicity of EMSI makes it an attractive alternative for prognostic evaluation in settings where NIHSS is not routinely performed. Emergency medical teams or prehospital personnel could rapidly assess EMSI to stratify patients early and guide treatment decisions before hospital arrival. Future research should focus on multicenter validation of EMSI as a prognostic tool to ensure its applicability across different healthcare settings. Prospective studies with longer follow-up periods will help clarify the trajectory of muscle strength recovery. Mechanistic studies should explore the biological underpinnings of rapid strength improvement, identifying factors that contribute to better outcomes. Additionally, the development of a predictive model that integrates EMSI with neuroimaging biomarkers may enhance accuracy in prognosis and guide targeted interventions.

While EVT has been established as a gold-standard treatment for AIS patients, its strength and limitations should be considered. It is worth emphasizing that for selected patient populations, EVT alone can be as effective as combining EVT with intravenous thrombolysis (IVT). A large-scale, multicenter study of 670 patients comparing 335 patients undergoing EVT alone with 335 patients receiving both EVT and IVT showed that no additional benefit to EVT was provided by adding IVT in AIS patients due to medium and distal vessel occlusion.^[22]^

Similarly, another study assessed EVT efficacy in patients with isolated M2 occlusion and minor stroke symptoms, aiming to identify baseline predictors of clinical outcomes.^[23]^ Data from 16 high-volume stroke centers on patients with a National Institutes of Health Stroke Scale (NIHSS) score of ≤5, who received either early EVT or best medical management with optional rescue EVT upon neurological deterioration, revealed no significant differences in clinical outcomes or safety between the early EVT and best medical management groups. Furthermore, no advantage was observed when comparing early EVT to rescue EVT. These findings suggest that in minor stroke patients with isolated M2 occlusion, early EVT does not offer added benefit over conservative management.^[23]^

In contrast, other studies showed that some complications can arise due to EVT.^[24,25]^ While functional outcome did not improve in AIS patients with distal medium vessel occlusion undergoing EVT in comparison to the best medical management in a large multicenter study of 2125 patients, EVT was associated with elevated hemorrhage.^[25]^ Therefore, care should be taken in using EVT patients with distal medium vessel occlusion.

Furthermore, first-pass effect (FPE) can be another factor influencing the outcome of AIS patients receiving EVT. Multiple factors, such as age, sex, diabetes mellitus, challenging arterial anatomy, and occlusion location have been reported as important predictors of FPE.^[26–28]^ PPE, in turn, can predict favorable outcome, reduce intracranial hemorrhage and 90-day mortality.^[29]^ Similarly, multiple other factors, including baseline and 7-day NIHSS, early neurological improvements have been reported as predictor of functional improvements following EVT.^[24,30–32]^

## 5. Limitations

Our study had some limitations which need to be acknowledged. This was a single-center study, with a limited sample size. Future multicenter studies with larger sample sizes are required to confirm the generalizability of our findings in different healthcare systems and populations. Differences in healthcare infrastructure, EVT protocols, and patient demographics may influence outcomes, making external validation essential.

The retrospective nature of this study introduces potential selection bias. While patients with missing follow-up data were excluded to maintain data accuracy, this could have skewed the results. A prospective cohort study is needed to validate our findings. Future studies should incorporate a multivariate regression model that accounts for additional prognostic factors such as post-EVT complications, rehabilitation intensity, and stroke subtype variability to further refine predictive accuracy. Despite these limitations, we believe that our study supports the use of early muscle strength assessment and encourages future research focusing on early muscle strength as a predictor of ischemic stroke patient outcomes. Future research should focus on multicenter validation of EMSI as a prognostic tool to ensure its applicability across different healthcare settings. Prospective studies with longer follow-up periods will help clarify the trajectory of muscle strength recovery. Mechanistic studies should explore the biological underpinnings of rapid strength improvement, identifying factors that contribute to better outcomes. Additionally, the development of a predictive model that integrates EMSI with neuroimaging biomarkers may enhance accuracy in prognosis and guide targeted interventions.^[33]^

## 6. Conclusions

In conclusion, EMSI has a similar predictive value as early NIHSS improvement for long-term outcomes after EVT. This study demonstrates that EMSI and the degree of muscle strength can be promising predictors of functional outcomes in patients with LVO stroke following EVT.

## Acknowledgments

We would like to thank all colleagues for data collection and the patients for their contributions.

## Author contributions

**Conceptualization:** Xuxing Liao.

**Data curation:** Zile Yan.

**Formal analysis:** Zile Yan.

**Investigation:** Zile Yan, Xuehua Zeng, Jiale Wu, Baoxin Chen, Zhiyi Zeng, Jianfang Gao, Rongshen Yang, Shuiquan Yang.

**Methodology:** Zile Yan.

**Supervision:** Shuiquan Yang, Xuxing Liao.

**Writing – original draft:** Zile Yan, Mohammad Mofatteh, Xuehua Zeng, Jiale Wu, Baoxin Chen, Zhiyi Zeng, Jianfang Gao, Rongshen Yang, Shuiquan Yang, Xuxing Liao.

**Writing – review & editing:** Zile Yan, Mohammad Mofatteh, Thanh N. Nguyen, Robert W. Regenhardt, Suzuki Dharmarasu, Shuiquan Yang, Xuxing Liao.
